# Correction: Highly sensitive and selective colorimetric detection of dual metal ions (Hg^2+^ and Sn^2+^) in water: an eco-friendly approach

**DOI:** 10.1039/d2ra90034c

**Published:** 2022-04-11

**Authors:** Rintumoni Paw, Moushumi Hazarika, Purna K. Boruah, Amlan Jyoti Kalita, Ankur K. Guha, Manash R. Das, Chandan Tamuly

**Affiliations:** Natural Product Chemistry Section, CSIR-North East Institute of Science and Technology Itanagar Arunachal Pradesh-791110 India c.tamuly@gmail.com; Dept of Chemistry, Cotton University Guwahati Assam-781001 India; Academy of Scientific and Innovative Research (AcSIR) Ghaziabad-201002 India; Material Science and Technology Division, CSIR-North East Institute of Science and Technology Jorhat Assam-785006 India

## Abstract

Correction for ‘Highly sensitive and selective colorimetric detection of dual metal ions (Hg^2+^ and Sn^2+^) in water: an eco-friendly approach’ by Rintumoni Paw *et al.*, *RSC Adv.*, 2021, **11**, 14700–14709, DOI: 10.1039/D0RA09926K.

The authors regret that the one of the affiliations (affiliation *c*) was incorrectly shown in the original manuscript. The corrected list of affiliations is as shown here.

The Royal Society of Chemistry apologises for these errors and any consequent inconvenience to authors and readers.

## Supplementary Material

